# Retroperitoneal Biloma Secondary to Operative Common Bile Duct Injury

**DOI:** 10.1155/1991/39181

**Published:** 1991

**Authors:** Radoje Čólović, Mirjana Perišić-Savić

**Affiliations:** Institute of Digestive Diseases University Clinical Center Medical Faculty Belgrade Serbia and Montenegro

## Abstract

Encapsulated collections of bile (“biloma”) may be a sequela of liver trauma, operative injury or disease. Such collections may be intrahepatic or extrahepatic and usually in the supramesocolic compartment of the abdomen. This is a report of a retroperitoneal biloma, an entity that has been reported only twice to date but this is the first secondary to an operative common bile duct lesion.

Evacuation of the biloma and reconstruction of the associated biliary stricture were successfully carried out. The patient remains sympton free with normal clinical and laboratory data more than 14 months after surgery.

Operative common bile duct (CBD) injury may be followed by a number of complications. To our knowledge retroperitoneal biloma secondary to a CBD lesion has not been previously reported.


*HPB Surgery*, 1991, Vol. 3, pp. 193-197
Reprints available directly from the publisher
Photocopying permitted by license only

© 1991 Harwood Academic Publishers GmbH
Printed in the United Kingdom

### CASE REPORT

### RETROPERITONEAL BILOMA SECONDARY TO OPERATIVE COMMON BILE DUCT INJURY

RADOJE ČÓLOVIĆ and MIRJANA PERIŠIĆ-SAVIĆ
*Institute of Digestive Diseases, University Clinical Center Medical Faculty Belgrade, Yugoslavia*

*(Received 15 August 1990)*

Encapsulated collections of bile ("biloma") may be a sequela of liver trauma, operative injury or disease. Such collections may be intrahepatic or extrahepatic and usually in the supramesocolic compartment of the abdomen. This is a report of a retroperitoneal biloma, an entity that has been reported only twice to date but this is the first secondary to an operative common bile duct lesion.

Evacuation of the biloma and reconstruction of the associated biliary stricture were successfully carried out. The patient remains sympton free with normal clinical and laboratory data more than 14 months after surgery.

Operative common bile duct (CBD) injury may be followed by a number of complications. To our knowledge retroperitoneal biloma secondary to a CBD lesion has not been previously reported.

### CASE REPORT

A 54 year old lady was submitted to elective cholecystectomy in a district hospital on 15/09/1988. Operative cholangiography was not performed. Soon after operation bile drainage through a subhepatic drain appeared and continued for four weeks. The patient subsequently developed bile peritonitis and was reoperated upon on 21/10/1988 and the abdominal cavity was drained. Bile drainage stopped by mid-January 1989 but was followed by jaundice. A third operation was performed on 23/02/89 for obstructive jaundice. The CBD could not be found and the abdomen was closed with drainage. In March 1989 percutaneous transhepatic cholangiography (PTC) was carried out clearly demonstrating a high CBD stricture. The procedure was followed by frequent attacks of severe cholangitis with a temperature of up to 40°C.. Once while vomiting she felt "that something broke in the abdomen". After this episode the fever abated somewhat and the attacks of cholangitis became less severe. However, a painful enlarging right lower quadrant abdominal mass appeared.

At the time of admission to our Institution on 31/03/1989 she was deeply jaundicied with a large tender right lower abdominal mass. Laboratory data indicated biliary obstruction (total serum bilirubin 302.5 mmol/l, serum alkaline phosphatase 6.9 U/l (normal value 1 to 2.5 U/l).

Ultrasonography (US) showed mild hepatomegaly and dilated intrahepatic bile ducts. The CBD was not visible. Below the liver in the retroperitoneum extending downwards almost to the pelvis a large septate encapsulated anechoic fluid collection 15 × 15 cm in diameter was demonstrated (Figures 1, 2, 3). The pancreas, spleen and kidneys were normal.

At operation on 03/04/1989 a CBD stricture (Type III Bismuth) was found with an internal biliary fistula extending along the hepatoduodenal ligament and behind the duodenum into the retroperitoneal space. A huge 1000 ml collection in the retroperitoneal space and mesenteric base was identified. The collection was emptied by an incision lateral to the ascending colon and the cavity was drained. The stricture was repaired using a Roux-en-Y hepatico-jejunostomy.

Recovery was uneventful. The serum bilirubin fell within the normal range within four weeks. Tube cholangiography through a transjejunal drain taken two weeks after operation showed free flow through a wide stoma into the jejunal limb. She remains sympton free with normal laboratory data, US and HIDA-scanning.

### DISCUSSION

Whipple in 1898[1] was the first to describe a cystic swelling containing bile stained fluid following liver trauma. Subsequently intrahepatic biliary cysts were reported [2-5] but are a very rare entity. Gould [6] and Patel 1979 described an encapsulated bile collection in the right-upper abdominal quadrant following blunt abdominal trauma and called it a "biloma". This term has been accepted by others. The size of reported bilomas vary from a few to 40 cm in diameter. The largest containing 5700 ml of bile [7]. Almost all reported bilomas occur as a result of blunt abdominal trauma [8] and are due to direct disruption of bile ducts, liver rupture with disruption

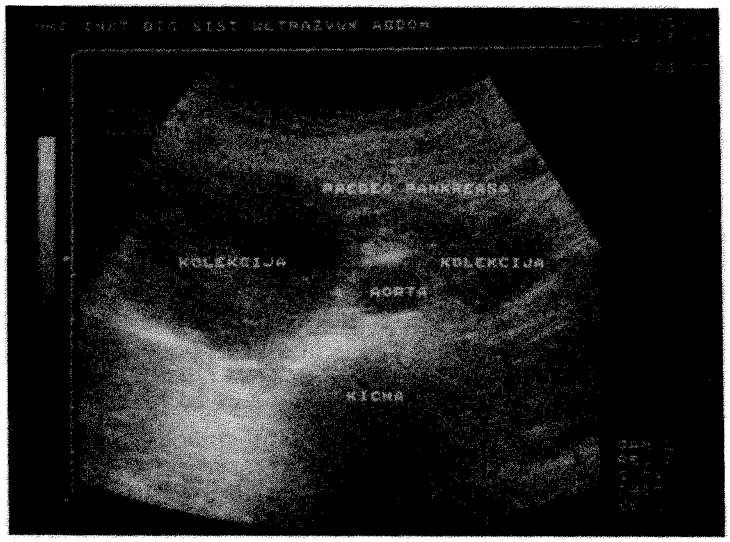

**Figure 1.** US showing retroperitoneal fluid collections, relation to pancreas and abdominal aorta.

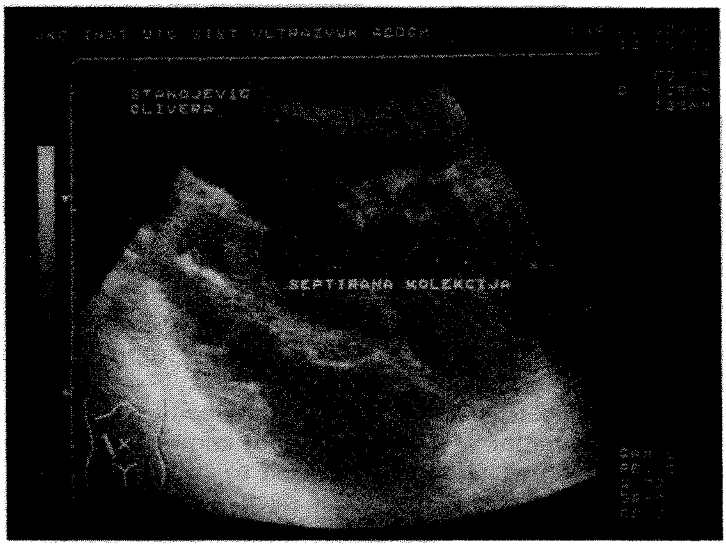

**Figure 2.** US showing a huge septated retroperitoneal fluid collection in the right abdomen, 15 × 15 cm in diameter.

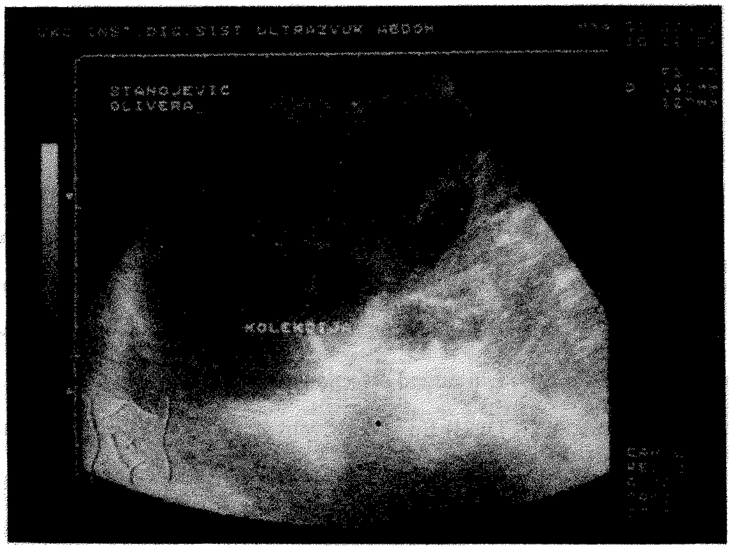

**Figure 3.** US showing the same fluid collection, extending downwards almost to the entrance of the pelvis.

of intrahepatic bile ducts or secondary tissue necrosis with bile leakage [9]. Rarely there is a spontaneous small perforation of the CBD or gallbladder with pericholecystic biloma formation [10]. Sometimes bilomas may develop after cholecystecomy [7,9], after invasive diagnostic procedures such as liver biopsy [11], PTC [9,12], percutaneous transhepatic drainage [9], ERCP [7] or endoscopic sphincterotomy [13]. It is possible that the pathogenesis of retroperitoneal biloma in our patient occurred during violent vomiting causing a rise of the abdominal pressure resulting in internal biliary leakage and temporary relief of cholangitis but giving rise to a retroperitoneal "biloma".

In all reported cases bilomas were located in the upper part of the abdomen [9,10], rarely in the left diaphragmatic area and are very rarely intrahepatic [1,12,14,15].

Neoptolemos *et al.* 1984 [13] reported a case of retroperitoneal bile leakage, abdominal wall bile staining and "biliscrotum" due to a retroperitoneal perforation of the CBD following endoscopic sphincterotomy.

A further case of retroperitoneal biloma was described by Satake *et al.* 1985 [16], due to spontaneous perforation of the CBD. To our knowledge the case presented here is the third retroperitoneal biloma reported to date but the first such case following operative injury to the common bile duct.

Symptoms of biloma may be absent, mild of severe and may appear at even a late stage. In our patient obstructive cholangitis and a painful growing abdominal mass were the leading symptoms. Biloma may be diagnosed by US [6,14,17,18]. Other diagnostic procedures may also be useful as for example hepatic scintigraphy (HIDA) [11,19,20], (CT-scan) [21,22].

Treatment may be operative or non-operative. Vujic and Brock 1981 [23] reported non-operative treatment with percutaneous, ultrasonic or CT guided aspiration of biloma. Others have confirmed the value of non-operative management [9,10,24,25]. However, in our patient surgery was necessary since reconstruction of the CBD for stricture was also required.

### *References*

1. Whipple, C. (1898) A case of traumatic rupture of the liver: formation of cystic swelling containing bile stained fluid. *Lancet* **1**, 719
2. Doran, A.H.G. (1903) Large bile cyst of the liver; jaundice without cholelithiasis. Incision and drainage recovery. *Lancet* **1**, 1218–1220
3. Henson, S.W. Jr. Hallenbeck, G.A., Gray, H.K. and Dockerty, M.B. Benign tumors of the liver. *Surg. Gynecol. Obstet.* (1957) **104**, 302–306
4. Sanfelippo, P.M. Beahrs, O.H. and Weiland, L.H. (1974) Cystic disease of the liver. *Ann. Surg.* **179**, 922–925
5. Jones, W.L. Maintain, J.C. and Warren, K.W. (1974) Symptomatic non-parasitic cysts of the liver. *Br. J. Surg.*, **61**, 118–123
6. Gould, L. and Patel, A. (1979) Ultrasound detection of extrahepatic incapsulated bile: "biloma". *AJR*, **132**, 1014--1015
7. Novis, B.H. and Adam, Y.G. (1982) Endoscopic retrograde cholangiography in case of biliary pseudocyst. *Endoscopy*, **14**, 24–25
8. Tucker, J.K., Everett, W.G. and Smith, J.M. (1975) Roux loop drainage for post-traumatic biliary cyst. *Br. J. Surg.*, **2**, 22--24
9. Mueller, P.R., Ferrucci, J.T. Jr. Simeone, J.F., Cronan, J.J., Wittenberg, J., Neff, C.C. and Sonnenberg, van E. (1983) Detection and drainage of bilomas: special considerations. *AJR*, **140**, 715–720
10. Vasquez, J.L., Thorsen, M.K., Dodds, W.J., Quiroz, F.A., Martinez, M.L., Lawson, T.L., Stewart, E.T. and Foley, W.D. (1985) Evaluation and treatment of intraabdominal bilomas. *AJR*, **144**, 933–938

11. Grijm, R., Tytgat, G.N. Schoot, van der J.B. and Royen, van E. (1984) Bile leakage after liver biopsy and its diagnosis by HIDA-scan. *The Netherlands J. Med.*, **27**, 408–411
12. Vujic, I., Meredith, H.C. and Anderson, M.C. (1979) A huge bile cyst-an unusual complication of percutaneous transhepatic cholangiography (PTC). *Australasian Radiol.*, **23**, 113–116
13. Neoptolemos, J.P., Harvey, M.H., Slater, N.D. and Carr-Locke, D.L. (1984) Abdominal wall bile staining and "biliscrotum" after retroperitoneal perforation following endoscopic sphincterotomy. *Brit. J. Surg.* **71**, 684
14. Esensten, M., Ralls, P.W., Colletti, P. and Halls, J. (1982) Posttraumatic intrahepatic biloma: sonographic diagnosis. *AJR*, **140**, 303–305
15. Charters, A.C. and Bardin, J. (1978) Intrahepatic bile duct rupture following blunt abdominal trauma. *Arch. Surg.*, **113**, 873–876
16. Satake, K. Ikehara, T., Shim, K., Tsuyoshi, A., Umeyama, K., Yoshioka, M. and Takemura, H. (1985) An incapsulation of bile from a spontaneous perforation of the common bile dict. *Am. J. Gastroenterol.* **80**, 279–283
17. Zegel, H.G., Kurtz, A.B., Perlmutter, G.S. and Goldberg, B.B. (1981) Ultrasonic characteristics of bilomas. *JCU,* **9**, 21–24
18. Ralls, P.W., Eto, R., Quionn, M. and Boger, D. (1981) Gray-scale ultrasonography of a traumatic biliary cyst. *J. Trauma* **21**, 176–177
19. Weissmann, H.S., Chun, K.J., Frank, M., Koenigsberg, M., Milstein, D.M. and Freeman, L.M. (1979) Demonstration of traumatic bile leakage with cholescintigraphy and ultrasonography. *AJR*, **133**, 843–847
20. Kuni, C.C., Klingensmith, W.C. III, Koep, L.J. and Fritzberg, A.P. (1980) Communication of intrahepatic cavities with bile ducts: demonstration with Tc-99m-diethyl-IDA imaging. *Clin. Nucl. Med.* **5**, 349–351
21. Kuligowska, E., Schlesisnger, A., Miller, K.B., Lee, V.W. and Grosso, D. (1983) Bilomas: A new approach to the diagnosis and treatment. Gastrointest. Radiol., **8**, 237–243
22. Frick, M. and Feinberg, S.B. (1982) Deceptions in localizing extrahepatic right-upper-quadrant abdominal masses by CT. *AJR,* **139**, 501–504
23. Vujic, I. and Brock, J.G. (1982) Biloma: aspirations for diagnosis and treatment. *Gastrointest. Radiol.* **7**, 251–254
24. VanSonnenberg, E., Ferruci, J.T Jnr, Mueller, P.R., Wittenberg, J. and Simeone, J.F. (1982) Percutaneous drainage of abscesses and fluid collections: technique, results and applications. *Radiology,* **142**, 1–10
25. Toriumi, D., Ruchin, M., Goldberg, M., Velasco, J., Silver, J., Berkowitz, G. and Atlas, B. (1989) Transnasal biliary drainage for treatment of common bile duct leakage and bile peritonitis. *Dig. Dis. Sciences* **34**, 315–319

(Accepted by S. Bengmark 27 August 1990

